# Effectiveness of Different Chemotherapeutic Agents for Decontamination of Infected Dental Implant Surface: A Systematic Review

**DOI:** 10.3390/antibiotics11050593

**Published:** 2022-04-28

**Authors:** Chayya Patil, Amit Agrawal, Shahabe Saquib Abullais, Suraj Arora, Shafait Ullah Khateeb, Mohamed Fadul A. Elagib

**Affiliations:** 1 Department of Periodontics, M.G.V’s K.B.H Dental College and Hospital, Nasik 422022, India; drcp0501@gmail.com (C.P.); agrodent@rediffmail.com (A.A.); 2 Department of Periodontics and Community Dental Sciences, King Khalid University, Abha 61421, Saudi Arabia; mfdel@kku.edu.sa; 3 Department of Restorative Dental Sciences, King Khalid University, Abha 61421, Saudi Arabia; surajarorasgrd@yahoo.co.in (S.A.); skhateeb@kku.edu.sa (S.U.K.)

**Keywords:** decontamination, peri-implantitis, peri-implantitis treatment, dental implant, biofilm removal, implant surface, chemotherapeutic agents, chemical decontamination

## Abstract

Aim: To evaluate the most effective chemotherapeutic agent for decontamination of infected dental implants. Material and methods: A systematic electronic literature search in MEDLINE (PubMed) and Google scholar between January 2010 to December 2021 was carried out by using the PRISMA guidelines. A total of five studies related to chemical decontamination of the dental implant were evaluated. The search strategy was based on the PICOS framework. Randomized controlled trials (RCT’s) and cohort studies evaluating the effectiveness of different chemotherapeutic agents for the decontamination of dental implants were included in the study. The outcome variable examined was the most effective chemotherapeutic agent(s) for dental implant surface decontamination after comparing the chemotherapeutic agents used in the qualifying studies. Result: Out of the basic database of 1564 records, 1380 articles were excluded due to irrelevance, unavailability, and repetition. Furthermore, 134 articles were excluded from 184 studies for various reasons. After further filtration, 13 studies were shortlisted. Two investigators (SSA and SA) appraised the quality of the selected studies using the risk of bias assessment tool. After excluding eight studies, five articles were finally included in the present systematic review. Conclusion: The data reported for the efficacy of chemotherapeutic agents in cleaning contaminated titanium surfaces are scarce, thus it is not possible to draw a definite conclusion. However, chlorhexidine (CHX) (0.2%, 0.12%), citric acid (40%) and sodium hypochlorite (1%) are the most commonly used chemotherapeutic agents; amongst them, citric acid showed the highest potential for biofilm removal from the contaminated implant surface. All three agents [CHX (0.2%, 0.12%), citric acid (40%), and sodium hypochlorite (1%)] can be recommended as therapeutic agents along with their curbs.

## 1. Introduction

Recently, oral implantology has become an integral part of dentistry. It helps dentists to improve the quality of life for a large patient population [1]. Dental implants are the best solution for simulating the aesthetic, perception, and function of natural teeth. Dental implants do not just replace the missing teeth, but also help in maintaining and reinforcing the bone structure. In a prospective cohort study, van Velzen et al. reported a 91.6% success rate for SLA-surface dental implants and reported 7% peri-implantitis after 10 years of follow-up [2]. Moraschini V et al., in their systematic review, evaluated the survival and success rate of osseointegrated implants for at least 10 years. Out of the final 23 articles selected, 14 studies showed survival rates of around 94.6% and mean marginal bone resorption of around 1.3 mm. They concluded that osseointegrated implants are safe and present high survival rates and minimal marginal bone resorption in the long term [3]. Atieh MA. systematically estimated the overall frequency of peri-implant diseases in general and high-risk group participants. The nine studies included showed that the frequency of peri-implant mucositis and peri-implantitis was 63.4% and 18.8%, respectively. Smokers showed a higher frequency (36.3%) of exhibiting peri-implantitis. They concluded that, to reduce the risk of peri-implantitis, long-term maintenance therapy for high-risk groups is essential, as peri-implant diseases are common after implant therapy [4].

Dental implants have a high success rate in the long term, however, failure is inevitable. Peri-implant disease, which is commenced by bacteria, has two subtypes: peri-implant mucositis, and peri-implantitis. At the first European workshop on periodontology (1993), peri-implantitis was defined as a destructive inflammatory process affecting the soft and hard tissues around osseointegrated implants, leading to the formation of a peri-implant pocket and loss of the supporting bone. Peri-implant mucositis was defined as “reversible” inflammatory changes in soft tissues surrounding a functional implant, without bone loss [5]. Reasons for implant failure, besides bacterial causes, are patient-related factors such as imbalanced occlusal forces, poor bone quality, improper surgical placement, poor oral hygiene, excessive surgical trauma, uncontrolled diabetes, smoking, bruxism, and edentulism status. [6].

Peri-implant tissues, similar to periodontal tissues, are susceptible to bacterial infection. Bacterial colonization of implant surfaces occurs rapidly. Fürst et al., in their study, have concluded that early colonization patterns differ between implant and tooth surfaces [7]. Bacterial diversity and its transition from a healthy peri-implant sulcus to an inflamed peri-implant pocket is related to bacterial shifts in dental plaque [8]. According to Quirynen et al., to improve or preserve periodontal health around titanium implants, a reduction of the number of pathogenic species is required [9]. Various methods were used for decontamination in previous studies, which are categorized into physical and chemical methods. Physical methods are further subdivided into mechanical and laser decontamination techniques. Photodynamic therapy falls into either category as it combines light-sensitive chemical agents with the laser used to promote their cytotoxicity. Chemical decontamination mainly involves the localized use of anti-microbial solutions such as topical chlorhexidine, tetracycline or minocycline, citric acid, hydrogen peroxide, 35% phosphoric acid gel, or saline [10]. Chemical decontamination can be combined with mechanical decontamination. Mechanical decontamination techniques alone were found to be not very effective in removing the bacterial biofilm, so adjunct use of different chemotherapeutic agents was advised to treat the infected dental implants [11].

A systematic review by Ntrouka VI et al., in search of the most effective chemotherapeutic agent for decontamination of the implant surface, has evaluated four different studies. They cautiously concluded that citric acid can be considered as a chemotherapeutic agent with the highest potential for the removal of biofilms from contaminated titanium surfaces in vitro. Yet, complete biofilm removal was not achieved by citric acid. They reported scarce data on this topic comparing the efficacy of chemotherapeutic agents on titanium implant surfaces [12].

In the present review, only five eligible studies were identified out of 1564 records obtained. Most of the studies have compared one chemotherapeutic agent with normal saline as a control group, so they were excluded as they failed to satisfy the inclusion criteria [13,14]. The systematic search was carried out to focus on “which is the most effective chemotherapeutic agent for decontamination of the infected dental implant (with or without adjunctive mechanical cleaning)?” using a structured search strategy and through the selection of the best available evidence. The purpose of this systematic review was to find the most effective chemotherapeutic agent for decontamination of infected dental implants (with or without adjunctive mechanical cleaning) from the existing literature.

## 2. Materials and Methods

This systematic review was conducted according to the guidelines of Preferred Reporting Items for Systematic Review and Meta-analysis (PRISMA statement 2009) [15,16]. This systematic review topic was registered on an international database of prospectively registered systematic reviews PROSPERO (CRD42020173838) to avoid any unintentional iteration/duplication of the review on the same topic.

### 2.1. Rationale and Focused Question for Review

To our knowledge, only one systematic review was carried out to explore the chemotherapeutic agents in the past literature including studies published on/before June 2010 [10]. There is no absolute explanation regarding the effectiveness of different chemotherapeutic agents in the decontamination of infected dental implants.

The addressed focused question is: “which is the most effective chemotherapeutic agent for decontamination of an infected dental implant (with or without adjunctive mechanical cleaning)?” In this review, an attempt has been made to answer this question using a structured search strategy and through the selection of the best available evidence.

To date, all of the studies and reviews have failed to distinguish a sole chemotherapeutic agent as the gold standard for implant surface decontamination [10,17]. Therefore, an effort was made to search the recent literature (from January 2010 to December 2021) focusing on the use and comparison of different chemotherapeutic agents for decontamination of infected implant surfaces. Thus, this review aimed to find evidence regarding the most effective chemotherapeutic agent for the decontamination of infected titanium surfaces (with/without mechanical decontamination).

### 2.2. Sources of Information and Search Strategy

A systematic electronic literature search in MEDLINE (PubMed) and the Google Scholar search engine was conducted. The separate search strategies were framed and used for the said databases. The search was limited to studies involving humans, in the English language, and published from January 2010 to December 2021.

A random combination of the following terms was used for the search: “peri-implantitis treatment”, “chemotherapeutic agents”, “implant surface decontamination”, “chemical disinfectant”, “titanium”, and “biofilm”. All retrieved articles were reviewed to identify additional relevant randomized controlled trials (RCTs). The titles and abstracts of potential references were manually examined to exclude irrelevant publications. Two reviewers independently reviewed all additional pertinent studies of the remaining pieces of literature on the topic of interest (Table 1).

### 2.3. Study Selection Criteria

In the current review, RCTs and cohort studies were included.

### 2.4. Eligibility Criteria

The current systematic review included in vitro and in vivo studies that evaluated the PICO question described above, excluding case studies or case series, animal studies, and literature reviews.

### 2.5. Primary and Secondary Outcomes

The primary outcome of this review was to identify the most effective chemotherapeutic agent(s) for dental implant surface decontamination after comparison among existing chemotherapeutic agents in the studies qualifying the inclusion criteria.

### 2.6. Screening and Data Extraction

After reading the title and abstract of initially identified studies from electronic databases, two reviewers (CP and AA) independently screened all the collected studies. Any duplication or articles that did not meet inclusion criteria were exempted. Studies with the full text available and meeting all the inclusion criteria were again assessed separately by the reviewers to determine whether they qualified the inclusion norms. Any disagreement/discrepancy was resolved by discussion. Once the screening was done, data were extracted by two independent authors from the relevant studies. Characteristics that have been extracted from each study were as follows: (i) year of publication; (ii) last name of first author; (iii) study type; (iv) sample size; (v) implant surface used; (vi) in-surgical or non-surgical; (vii) with or without mechanical cleaning; and (viii) mode of application of the chemotherapeutic agent.

### 2.7. Quality Assessment

Two review authors (CP and SSA) independently assessed the quality of each study and the risk of bias in the included studies. After a preliminary evaluation of the selected papers, considerable heterogeneity was found in the study designs, characteristics, outcome variables, and measurements.

### 2.8. Statistical Analysis

Meta-analysis was not carried out due to the heterogeneity of data.

## 3. Results

**Figure 1:**
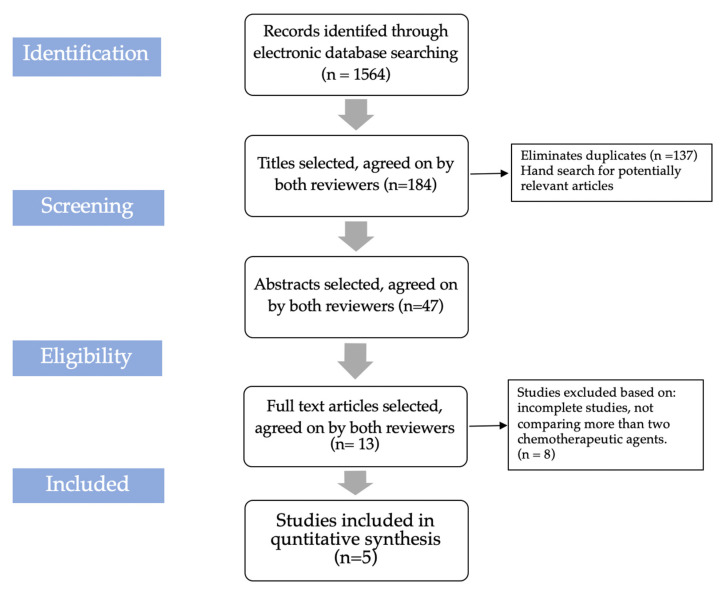
Literature search flowchart.

A total of 1564 records were identified through the database searches (PubMed, Google Scholar), out of which 1380 records were excluded as they were irrelevant, as data units were unavailable, or due to repetition. The remaining 184 articles were assessed for eligibility based on their title, out of which 134 articles were excluded due to the following reasons: not relevant to implant surface decontamination; articles not in English; and articles in which other methods were used for implant surface decontamination. Furthermore, out of the 47 remaining articles, 13 were selected based on the keywords such as “peri-implantitis treatment”, “chemotherapeutic agents”, “implant surface decontamination” and “chemical disinfectant for implant surface”. Thirty-four full-text articles were excluded for the following reasons: comparison between different decontamination methods; and comparison of only one chemotherapeutic agent with normal saline as the control group. In the final step of article selection, out of 13 articles, eight were excluded due to incomplete studies, not comparing more than two chemotherapeutic agents. Thus, finally, five studies were included in the present systematic review. The selection process was outlined in the PRISMA flow chart (Figure 1). Details of the selected studies are summarized in Table 2.

Risk of bias assessment:

There could be a potential language bias in this systematic review as we only considered literature written in English. Two investigators (AA, SSA) appraised the quality of the selected studies separately using the risk of bias assessment tool (The Cochrane collaboration’s tool) [18]. If there was any debate over a review, then it was settled by conversation. By using the risk-of-bias assessment tool, the studies were categorized as high, low, or unclear risk of bias. After the quality assessment, the included studies were graded into (1) low risk: when all criteria were met, or one criterion was unclear/not met; (2) moderate risk: when two criteria were unclear/not met; (3) high risk: when more than two criteria were not met. There are 13 studies in which different chemotherapeutic agents were compared on contaminated implant surfaces. Out of these, nine studies showed heterogeneity in terms of contamination, different chemicals used for decontamination in different studies, and outcome measures.

**Table 2:** Details of the selected studies for systematic review.

Author (Year, with/without Mechanical Cleaning)	Implant Surface Contaminated with	Chemical Used (Application Form, Time)/Type of Intervention	Sample Size	Outcome Measure/Primary Endpoint	Conclusion/Outcome
Gosau M et al. [19] (2010, without)in vivo	Oral biofilm	1% sodium hypochlorite (NaOCL) 3% H_2_O_2_0.2% CHX gluconate Plax (Triclosan 0.3%) Listerine cool mint (alcohol based) Citric acid (Ph 1, 40%) PBS solution(control) (in liquid form, for 1 min)	8 8 8 8 8 8 8	The proportion of live/dead bacterial cell	All six antimicrobial agents were effective in reducing oral bacterial biofilm on titanium disc, compared to control. All (except Plax, 40%) showed a significant bactericidal effect on adhering bacteria.
Ntrouka et al. [20] (2011, without) In-vitro	1-streptococcus mutans	24% EDTA 40% Citric acid 10% H_2_O_2_ Ardox-X 0.07% CPC 0.2% CHX digluconate Sterile water(control) (in liquid form, for 5 min)	6 6 6 6 6 6 6	1. Total CFU count 2. Protein measurement (µg)	H_2_O_2_, Ardox-X, 40% citric acid Were most effective & Ardox-X, 40% citric acid were most potent in killing streptococcus mutans. 40% citric acid was most effective in bacterial killing, the addition of H_2_O_2_,/Ardox-X to Citric acid didn’t have a significant effect
	2-saliva to grow polymicrobial biofilm	40% Citric acid (5 min) Ardox-X (5 min) 10% H_2_O_2_ (5 min) Ardox-X then Citric acid (2.5 min each) 10% H_2_O_2_ then Citric acid (2.5 min each)	6 6 6 6 6		
R Burgers et al. [21] (2012, without) In-vitro	Staphylococcus epidermis	1% sodium hypochlorite 3% H_2_O_2_0.2% CHX gluconate Plax (Triclosan 0.3%) Listerine Citric acid (Ph 1, 40%) Saline(control) (in liquid form, for 60 s) (The chemicals used are not categorized well please arrange)	35	The proportion of live/dead bacterial cell	Only sodium hypochlorite (1%) was effective against all 3 species. Whereas H_2_O_2_ only against Candida albicans. CHX gluconate (0.2%) & Listerine against Candida albicans and Streptococcus sanguis. Plax (0.3%) against Streptococcus sanguis and Staphylococcus epidermis.
	Candida albicans		35		
	Streptococcus sanguis		35		
Georgis A Kotsakis et al. [22] (2016, without)	Multi-species biofilm	0.12% chlorhexidine 20% citric acid gel 25% EDTA 15% sodium hypochlorite 0.9% NaCl (sterile saline) (Burnished for 20 s with cotton pellet moistened in chemical agent	6 6 6 6	1. CFU count 2. Surface characterization	Antimicrobial effect was greater for citric acid, sodium hypochlorite/EDTA groups followed by Chlorhexidine (0.12%) group as compared to non-contaminated control. sterile saline only had a minimal antimicrobial effect. Chlorhexidine (0.12%) use is not recommended as it produces a cytotoxic effect on the decontaminated surface and compromise the biocompatibility of the titanium surface.
Dostie S et al. [23] (2017, without)	Multi-species mature oral biofilm	Control group (not rinsed/treated with chemical)	3	1. Bacterial cell count 2. Viability of bacteria after treatment	The double rinse group removed more bacteria compared to the rinse group. But no significant difference between the double rinse and disinfectant group suggests a mechanical effect of rinsing was responsible for the removal of bacteria and not the chemical effect. Proportion of dead cells for CHX group (11.8%), Etch group (6.9%) & tetracycline (3.9%) respectively compared to double saline group. No significant difference was noted between double rinse and the C.C.E. group.
		Rinse group (0.9% sodium chlorite, 6 increments, total 6 mL)	3		
		CHX group (1% Chlorhexidine in methylcellulose gel)	3		
		Etch group (35% phosphoric acid gel)	3		
		Tetracycline group (250 mg tetracycline with 0.9% NaCl to form thick paste)	3		
		C.C.E group (0.3% cetrimide, 0.1% CHX, 0.5% EDTA in 3% methylcellulose gel)	3		
		Double-rinse group (12 increments of 1 mL 30.9% NaCl) without any chemical agents (Irrigation/application of gel, 2 min)	3		

## 4. Discussion

The dental implant has become a crucial therapy in dentistry to replace missing teeth in different clinical situations. Once the biofilms are completely formed on implant surfaces, the peri-implant tissue response seems to follow patterns similar to those of the periodontal tissues in a susceptible host. Over the last few decades, there is increasing evidence of the presence of peri-implant inflammation representing one of the most frequent complications affecting both the surrounding soft and hard tissues, which can lead to implant failure. Therefore, strategies for the prevention and treatment of peri-implant disease should be integrated into modern rehabilitation concepts in dentistry. Peri-implant inflammation represents a serious matter after dental implant placement, which affects both the surrounding hard and soft tissue [24]. Despite various preventive and treatment modalities for the treatment of peri-implant diseases, none has shown excellent results. Chemical surface decontamination is one of the treatment modalities in which different chemotherapeutic agents are used for surface decontamination.

Persson et al., in an animal study used two-part implants. Peri-implantitis was induced on those parts. Complete re-osseointegration was observed after decontamination. So, they insisted that decontamination of the titanium surface is of utmost importance for re-osseointegration [25]. Leonhardt A et al., in their 5-year follow-up human study evaluated the outcome of combined surgical and antimicrobial treatment of peri-implantitis. After surgical exposure, the implant surface was treated with 10% hydrogen peroxide, and washing with saline solution was carried out. It was followed by a 0.2% chlorhexidine mouthwash and systemic antibiotic therapy (amoxicillin + metronidazole). Results showed a significant reduction in plaque and gingival bleeding. Out of 26 implants included in the study, seven were lost within 5 years of follow-up, six showed increases in bone level, four showed a continued bone loss, and in nine, bone level was not changed significantly. Based on the clinical, microbiological, and radiographic evaluation they concluded a 58% success rate of implants after chemical decontamination [26]. In another study, it was noted that biofilm formation was increased during exposure to the oral environment, due to the implant surface morphology and roughness [27]. Thus, the effect of mechanical debridement was limited [28]. Kozlovsky et al. [29] and Renvert et al. [11] put forward the use of chemotherapeutic agents as an adjunct to mechanical therapy. As titanium surfaces are not instrumented in chemical decontamination, it led to minimal risk of damage [30].

Among all modalities, decontamination using chemotherapeutic agents is one of the promising treatment modalities to control the further progression of peri-implant disease. The past literature shows various studies on the chemical decontamination of titanium implant surfaces. In those studies, implant surfaces were contaminated either with specific microorganisms or with an oral biofilm, and different chemotherapeutic agents were used for decontamination. The outcome parameters evaluated in each selected study were different. Considering this heterogeneity of the studies in the literature, the results in this study were based on the most frequently used chemotherapeutic agents. In the five studies included in this review, the chemotherapeutic agents were chlorhexidine, citric acid, and sodium hypochlorite.

In a discourse on citric acid, Ntrouka VI et al. [10] carried out a systematic review in which they cautiously reported that citric acid is considered a most promising chemical agent with the highest potential for biofilm removal from titanium surfaces. This was mostly based on the findings of in-vitro studies. Lastly, they noted that complete removal of the biofilm was not achieved by any particular chemotherapeutic agent. Wheelis et al. [31] showed that citric acid leads to changes in the titanium surface, resulting in a greater roughness after treatment (which includes an increase in the depths of valleys and the prominence of peaks) when analyzed using the electron microscope. It was found that citric acid seems to dissolve the oxide layer when the acid is applied under abrasion. Also, the degradation of the implant oxide film can influence the electrochemical behavior of titanium, leading to its corrosion and favoring the release of particles and debris that may cause detrimental outcomes to surrounding tissue [31,32,33].

Souza et al. [14] in their study evaluated the antimicrobial effect of citric acid on in-situ biofilms, whether this treatment favors bacterial recolonization, and its effect on the electrochemical properties of titanium. Palatal appliances containing titanium discs were worn by volunteers for around 7 days. After 7 days, the discs were exposed to subsequent treatment: immersion in 0.9% sodium chloride (control); 40% citric acid immersion; and 40% citric acid rubbing. After chemical treatment, appliances were exposed in-vitro to new bacterial adhesion with Streptococcus sanguinis. New discs (*n* = 18) without a biofilm were exposed to the various treatments and subjected to electrochemical tests and surface characterization. Results showed that the citric acid groups had a significant reduction in the biofilm formed in-situ compared with the control group (*p* < 0.05), but no difference was found between the citric acid application methods (*p* = 0.680). The acid treatment did not favor the recolonization of bacteria (*p* = 0.629). Citric acid treatment did not influence the polarization resistance and capacitance of the oxide film, but statistically enhanced the electrochemical stability of titanium. Therefore, they conclude that citric acid can be used as an effective alternative to treat the main etiologic factor in dental implant failure, biofilm formation, without impairing the electrochemical behavior of the titanium surface.

Recently, in vitro studies on the toxicity of citric acid have been evaluated and it was found that citric acid at 4% to 10% concentrations did not result in cytotoxicity on human osteoblastic cells. A significant decrease in cell proliferation was reported, but it was also reported that the normal proliferation rate was restored around 3 days after treatment with a 4% concentration [34]. In the literature, mostly citric acid was used at pH 1 with a concentration of 40%. In an in-vitro study, it was found that citric acid suppressed the attachment and spreading of fibroblasts on culture plates and type-I collagen, along with confirming that the toxic effect of media containing citric acid was due to their acidity rather than the citrate content [35]. Thus, citric acid application must be limited to the implant surface. Clinical application of citric acid is more difficult because of the mandate to avoid the its spread to bone and marrow spaces.

In the literature, chlorhexidine was used for decontamination of contaminated titanium implant surfaces at 0.12% and 0.2% concentrations, mostly. Menezes KM et al., 2016, in a human study, evaluated the efficacy of 0.12% chlorhexidine gluconate in peri-mucositis patients. A total of 37 peri-mucositis patients were assigned to either the test group (basic periodontal therapy +0.12% chlorhexidine) and the control group (basic periodontal therapy+placebo). Clinical parameters were evaluated at baseline and one, three, and six months post-therapy. The results showed a statistically significant improvement in the plaque index, gingival bleeding index, probing depth, and bleeding on probing. They concluded that mechanical therapy alone, and along with 0.12% chlorhexidine mouthwash, reduced peri-implant mucositis [36].

In a study on canines, it was found that chlorhexidine has also been shown to favor re-osseointegration. In induced peri-implantitis lesions, the bony lesions were debrided post flap elevation and the implant surfaces were mechanically cleaned using curettes followed by a rinse with 0.12% chlorhexidine. Guided tissue regeneration (GTR) membranes were adapted and covered using flaps. Metronidazole was given to the animals. A total of 60 to 80% of bone fill was obtained and re-osseointegration ranged from 2 to 19.7% [37]. In another animal study, the implant surface was debrided and then rubbed with chlorhexidine soaked gauze followed by a rinse with saline approximately 20 times. Implants were randomized to receive autogenous bone grafts and platelet-enriched fibrin glue or just chlorhexidine. The combined treatment resulted in 50.1% re-osseointegration while the chlorhexidine group revealed a mere 6.5%. [38] In a recent study on monkeys, 0.1% chlorhexidine applied with gauze for 5 min, with a subsequent rinse using chlorhexidine and saline 20 times, yielded a 14% re-osseointegration. If the same therapy was given in combination with an autogenous bone graft, there was 22% re-osseointegration and an extended polytetrafluoroethylene (e-PTFE) membrane alone showed 21% re-osseointegration. However, chlorhexidine, bone graft, and membrane combination resulted in the highest rate of 45% [39].

In a randomized clinical trial, chlorhexidine was evaluated for a reduction in the total anaerobic bacterial load and putative periodontal pathogens. Forty-eight implants with peri-implantitis were debrided surgically and cleaned using gauze soaked in saline, with subsequent irrigation using a solution of 0.12% chlorhexidine plus 0.5% cetylpyridinium for 1 min, and then rinsed with saline. Irrigation with a placebo solution was done for the control group (31 implants). One-year follow-up showed no statistically significant difference between the groups in terms of bacterial counts or clinical markers like plaque, bleeding on probing (BOP), or suppuration [40]. The cell toxicity of chlorhexidine on human bone cells has been assessed. Cellular toxicity seems to be affected by concentration and exposure time. Scanning electron microscope (SEM) analysis proved the absence of osteoblast phenotypic alterations after exposure to 0.2% chlorhexidine for one minute and chlorhexidine 1% for 30 s [41]. Another in-vitro study demonstrated that chlorhexidine influenced osteoblast viability in a dose- and time-dependent manner. It resulted in apoptotic and autophagic/necrotic cell deaths and involved disturbance of mitochondrial function, intracellular Ca^2+^ increase, and oxidative stress. Chlorhexidine has also been shown to restrain cell proliferation and collagen synthesis [42,43].

Sodium hypochlorite (1%) shows broad antimicrobial activity, rapid bactericidal action, and relative non-toxicity at common concentrations [44]. It demonstrates high bactericidal and fungicidal activity in experimental biofilms with various endodontic or periodontal pathogens [44,45,46]. Subgingival irrigation with 0.5% sodium hypochlorite causes a significantly higher and long-lasting reduction in plaque and gingivitis than irrigation with water [45,47,48]. Thus, it was used as an effective decontaminating agent for infected titanium surfaces of implants. Gosau M et al. [19] concluded, along with other chemotherapeutic agents, that sodium hypochlorite is effective in reducing the oral bacterial biofilm on titanium disc as compared to the control. R. Burgers et al. [21] concluded that only sodium hypochlorite (1%) was effective against all three species of microorganisms, such as Staphylococcus epidermis, Candida albicans, and Streptococcus sanguis.

Retrograde peri-implantitis was also one of the reasons for implant failure, although its prevalence is very low (0.26%) [49]. Incidence increased up to 7.8% when adjacent teeth showed a previous history of root canal therapy [50]. It appears as a radiolucency around the most apical part of an implant. Different treatment modalities of retrograde peri-implantitis have been suggested to increase the survival of the involved implant. Among these modalities, Park et al. [51] suggested the use of systemic antibiotics plus 0.12% chlorhexidine mouthwash. In a case report, they surgically retrieved the retained root fragment and implant and replaced it with a new wide-body implant along with DFDBA mixed with tetracycline. At the end of 6 months follow-up, increased radiographic density, and bone formation were noted in one case, while in other cases, good defect repair was not noted. Thus, they concluded that antibiotics can be used for disease suppression until a definitive surgical procedure is performed. Soldatos et al., in their report, presented the comprehensive management of retrograde peri-implantitis by using an air-abrasive device, Er, Cr: YSGG, and guided bone regeneration (GBR). The site was monitored for 13 months and increased radiographic bone density was noted [52]. A recent literature review by Sarmast et al. focused on the novel treatment decision tree for retrograde peri-implantitis. They concluded that the most common etiology for retrograde peri-implantitis is endodontic infection from neighboring teeth, which was diagnosed within 6 months of the implant insertion. Common clinical and radiographic findings are sinus tract and radiolucency around the implant apex respectively [53].

Based on observed facts, most of the studies have different outcomes tested and it is inconsistent and unpredictable. The literature search showed there is still no consensus among clinicians regarding the best treatment. All the treatments described in past were effective with their pros and cons, but a systematic approach to the decontamination treatment of contaminated implant surfaces, along with more in vivo studies, should be initiated. Due to the heterogeneity of the data, it is challenging to categorize and evaluate the data in a controlled manner and finally come to any definitive conclusions, as no two studies showed the same type of chemotherapeutic agent used, similar bacterial contamination, and outcome parameters.

Limitations of this study were: the heterogeneity of the data; the limited number of studies with restricted data; not a single study comparing all the existing chemotherapeutic agents, comparing constant outcome measures, and the same type of titanium metal/implant surface; and differences in the microbial contamination of implant surface in all existing studies. Thus, there is a comparatively high risk of bias. Hence higher-quality RCTs comparing more chemotherapeutic agents with each other and normal saline, with a substantial sample size are needed to draw a definitive conclusion.

## 5. Conclusions

Within the limitations of the present study, it seems that chlorhexidine (0.2%, 0.12%), citric acid (40%), and sodium hypochlorite (1%) are the most commonly used chemotherapeutic agents in previous studies. They can be recommended along with their pros and cons for the decontamination of contaminated titanium implant surfaces. They are effective at killing bacterial cells, and biofilm removal, along with minimal/no alteration of the surface characteristics of the titanium implant surface.

However, only a preliminary conclusion can be drawn from this study due to the limited number of studies with restricted and heterogenous data. Thus, we conclude that the existing data is inconclusive.

## Figures and Tables

**Table 1:** Systematic search strategy.

I. Focus Question	“Which Is the Most Effective Chemotherapeutic Agent for Decontamination of an Infected Dental Implant (with or without Adjunctive Mechanical Cleaning)?”
II. Search strategy *P—Population* *I—Intervention* *C—Comparison* *O—Outcome*	*Infected dental implants/different chemotherapeutic agents?* *Effectiveness of different chemotherapeutic agents used for implant surface decontamination and comparison of them, with or without mechanical cleaning of the implant surface.* *Use of chemotherapeutic agents along with mechanical cleaning* *To identify the most effective chemotherapeutic agent (s) for dental implant surface.*
III. Search keywords	Peri-implantitis treatment, chemotherapeutic agents, implant surface decontamination, chemical disinfectant for implant surface.
IV. Database search	PubMed, Google
V. Selection criteria *Inclusion criteria* *Exclusion criteria*	*Studies involving a minimum of two chemotherapeutic agents for implant decontamination* *Contaminated implant surface* *Decontamination was done without implantoplasty* *Only in the English language* *Experimental human studies* *Where the full text is not available* *No access to an English version of the title and abstract.*

## Data Availability

The data presented in this study are available on reasonable request from the corresponding author. The data are not publicly available due to privacy restrictions.
